# The geographic variation and spatiotemporal distribution of hepatitis C virus infection in Libya: 2007–2016

**DOI:** 10.1186/s12879-018-3471-4

**Published:** 2018-11-22

**Authors:** Mohamed A. Daw, Lutfi A. Buktir Ali, Amina M. Daw, Nadia E. M. Sifennasr, Aghnyia A. Dau, Mohamed M. Agnan, Abdallah El-Bouzedi, In association with the Libyan Study Group of Hepatitis & HIV

**Affiliations:** 1 Department of Medical Microbiology &Immunology, Faculty of Medicine, University of Tripoli, CC 82668 Tripoli, Libya; 2 Department of Infectious Disease, Tripoli Medical Centre, Tripoli, CC 82668 Tripoli, Libya; 3 Department of General Medicine, Faculty of Medicine, University of Tripoli, CC 82668 Tripoli, Libya; 4 Department of Surgery, Tripoli Medical Centre, Faculty of Medicine, University of Tripoli, CC 82668 Tripoli, Libya; 5 Department of Toxicology, Faculty of Medical Technology, AlgabalAl-garbi University, Nalut, Libya; 6 Department of Laboratory Medicine, Faculty of Biotechnology, Tripoli University, CC 82668 Tripoli, Libya

**Keywords:** HCV infection, Libya, Geospatial analysis, HCV hotspots, Spatiotemporal distribution, Geographic variation

## Abstract

**Background:**

Hepatitis C Virus infection has been considered an important hidden pandemic in developing countries, particularly in Africa. It varies greatly from one region to another and even within districts of the same region. Macroscopic geospatial analysis has become an important scientific tool for identifying the density and clustering of HCV infection and provides epidemiological information for planning interventions and control strategies. The application of these parameters provides a better knowledge of the hepatitis C virus infection prevalence at the national level and can help to implement pertinent strategies to address the HCV-related burdens. This study aims to determine the geographical variability of HCV infection in Libya and to identify the hot spots within regions and districts of the country, and to analyze the population-based demographic determinants involved and outline the intervention programs needed.

**Methods:**

Disease mapping and spatial analysis were conducted using geographic information data available on all documented cases of HCV infections in Libya between 2007 and 2016. Spatial autocorrelation was tested using Moran’s Index, which determines and measures the degree of clustering and dispersion of HCV infection in a country.

**Results:**

A total 114,928 HCV infection cases during a ten-year period with accurate geographic information were studied. Ages ranged between 16 and 50 years and the male to female ratio was 2:1. HCV infection was unevenly distributed in Libya, and its incidence increased steadily over the study period**.** Severa**l** hot spots and cold spots were found mainly in the southern and eastern regions of the country.

**Conclusion:**

HCV infection in Libya was geographically variable, with several hot spots particularly in eastern and southern Libya associated with different demographic determinants. Future intervention planning should consider the geospatial variability and risk factors involved.

## Background

Hepatitis C virus (HCV) infection is an important burden for healthcare services worldwide. Over 32 million people are chronically infected with HCV in Southeast Asia and more than 6 million in Latin America. Also, sub-Saharan Africa houses almost 20% of world’s HCV-infected individuals [1, 2]. HCV-infected individuals have a 2.4 times higher risk of all-cause mortality compared to the non-infected population, 26.5 times the risk of liver-related mortality, and 1.8 times the risk of non-liver-related mortality [3, 4].

In North and sub-Saharan African countries, where HCV is considered endemic, its prevalence varies greatly among African countries [5]. It has recently been aggravated by immigration and massive population displacements caused by economic hardship and political instability. These factors could influence the geographical status of HCV infections, particularly in North African countries, which are transited by African migrants on their way to Europe [6].

Libya is the second largest country in North Africa and has the longest coast on the Mediterranean basin. It is surrounded by six countries where HCV is endemic, including Egypt, which has the highest prevalence of HCV in the world. Close proximity to these countries could provide transmission arteries for HCV infection into and across Libya [7, 8]. Furthermore, Libya is an oil-rich country that has for decades performed well economically and attracted many Africans as a place to work and as a route to Europe. In Libya, HCV prevalence has been reported to be relatively moderate at 1.2%, although it varied greatly from one region to another [9, 10]. Immigration is a particularly important factor in HCV prevalence in Libya. A recent study carried out on African immigrants residing in Tripoli showed a prevalence of HCV ranging from 2 to 26%, depending on the country of origin of the immigrants. The highest rate was among those from Egypt (18.7%) and West Africa (14.1%), followed by immigrants from the Horn of Africa and sub-Saharan countries [11]. Furthermore, the geographic variation of HCV genotypes in Libya varied from one region to another [12]. Genotype 4 was predominant, with a higher level in the eastern region, followed by genotypes 1 and 2, which were common in the western part of the country [12].

The World Health Organization recommends in its new guidelines that HCV tests be offered to people with high risk behavior and to those from high risk populations [13]. However, screening programs targeting these risk groups, and particularly intravenous drug users and promiscuous individuals, have not always been effective, particularly in developing countries at least partly due to stigmatization of such behaviors. Hence, specific studies, such as geospatial analysis, should be applied to determine clustering of HCV infections associated with socio-economic characteristics, level of urbanization, and population density [14–16].

Geospatial analysis is valuable in determining the spatial distribution and quantifying the impact of socio-economic determinants on the incidence of infectious diseases. In China, Europe and USA, such information has been widely used by public health services to identify areas of high risk for HCV, HIV and other concomitant infections [17, 18]. There is little information on the spatiotemporal dynamics of HCV, particularly in North and sub-Saharan Africa, where HCV infection is endemic [19–21].

A better understanding of the distribution of these diseases would provide a rational basis for allocating medical resources and providing effective guidance for public health interventions. The aims of this study were to determine the geographic variation and spatiotemporal distribution of HCV infection in Libya and to analyze the socio-economic and demographic determinants associated with the distribution of HCV infection.

## Methods

### Data source

In 2006, Libya carried out one of the largest nation-based surveillance studies of hepatitis B and hepatitis C viruses using a sample of > 1% of the total Libyan population [9]. Since then, all diagnosed cases of HCV infection are being reported and officially documented. The HCV screening policy and notification system in Libya is based on mandatory, anonymous notification of newly diagnosed HCV cases by laboratories all over the country combined with epidemiological information on the mode of transmission and other clinical data reported by physicians and trained clinical epidemiologists. The national screening policy for HCV in Libya, which is uniform all over the country, includes screening of blood donors, preoperative patients, pregnant women, and kidney and liver disease patients, as well as pre-marriage screening.

**Fig. 1:**
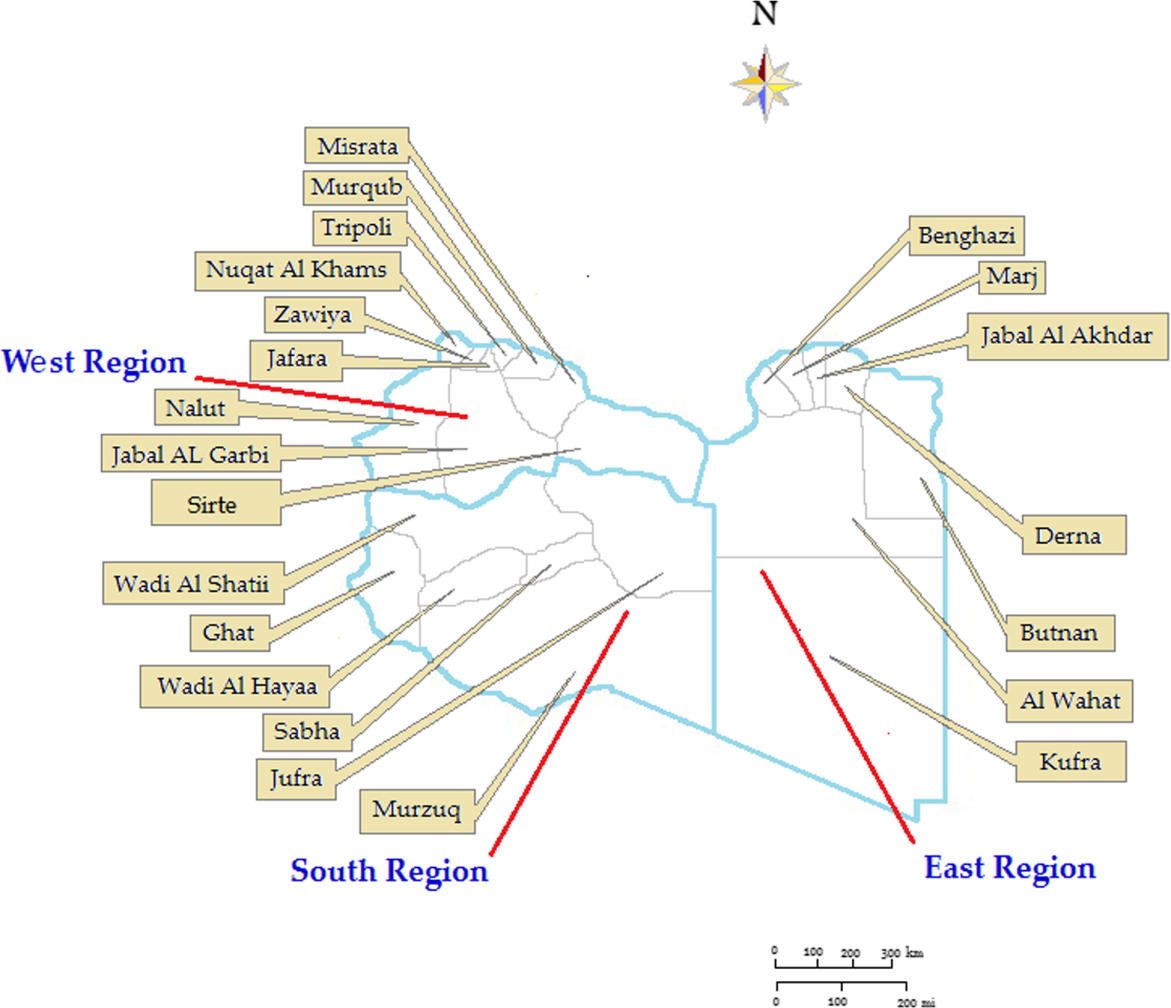
The geographical locations and boundaries of the Libyan regions and districts

The data collected so far consist of all the HCV infections newly reported throughout the country between January 2007 and December 2016 and include 114,926 individuals**.** The data covered all the Libyan regions and the residence of each case was localized at the city and district levels (Fig. 1).

Laboratory diagnosis was carried out according to the procedures of the Libyan Central Laboratory. HCV testing was carried out by detecting HCV antibodies using an enzyme-linked immunosorbent assay (ELISA) and confirmed by immunoblot and/or polymerase chain reaction. Any person found to be positive and confirmed by these diagnostic tests is considered to be a case of HCV infection [9, 11].

### Geospatial analysis and mapping of HCV infections

Geo-data (Libya’s map and the administrative municipalities’ geographical borders) and patient data were used for geospatial analysis on grid maps. Overall HCV prevalence was estimated based on the number of patients found infected and the total population of Libya and of each of its municipalities during the study. The locations of the infected individuals were marked at the district level on the most recently updated map of Libya showing its districts. On the maps were indicated the spatial mean center and spatial standard distance ±2 standard deviations.

The electronic maps were made with ArcGIS 10.1 software (Environmental Systems Research Institute (ESRI), Inc., 1999; Redlands, CA, USA) and the data were processed and analyzed with SPSS18.0 software (IBM Inc., Armonk, NY, USA).

### Spatial autocorrelation

Spatial analysis was carried out to identify the locations of HCV clusters and surrounding areas. To identify the spatial patterns of the infected cases, gobal Moran’s I statistic was used to analyze the presence and nature of HCV spatial autocorrelation (or dependency) in the original 22 districts for each year between 2007 and 2016 by using Open-GeoDa 1.2.0 [22, 23]. A Moran index value of − 1 indicates perfect dispersion, + 1 perfect correlation, and zero value random spatial distribution. The measures of spatial association were used to identify the locations of clusters and outliers and to determine the importance and the type of spatial correlation.

HCV clustering, which could be inferred from the presence of spatial autocorrelation, sometimes indicates hierarchical expansion in urban areas and across districts. A high frequency area means that it contained many HCV cases and that it was surrounded by regions also with a large number of cases. Conversely, a low frequency area means that it had few cases and was surrounded by areas also with few cases.

### Statistical analysis

The epidemiological data were collected using a standard registry form report, as previously described (12879_2013_2969_MOESM2_ESM.docx),(12879_2013_2969_MOESM1_ESM.doc).

The statistical analysis was performed using computer software version 11.0 (StataCorp. 2013. Stata Statistical Software: Release 13. College Station, TX: StataCorp LP). Logistic regression was conducted to identify the factors associated with HCV infection. Data were expressed as means ± standard deviation (SD), and as number and percentage with 95% confidence interval (CI), as appropriate. The z-test was used to determine if the rate ratio was statistically significant (*p* < 0.001) [9]. The adjusted relative risk (RR) of HCV infection comparing females to males was estimated. RR calculations were adjusted for variables known to be associated with HCV infection in Libya, such as social status, living conditions or demographic characteristics (age, gender, marital status and education, etc.)

## Results

A total of 114,928 newly reported HCV cases during a ten-year period with specific, accurate geographic information were included in this study. The geographic distribution of the reported HCV infection cases was found to vary between the Libyan regions and districts (Table 1). There were 53,358 cases in the western region (46%), 39,220 in the eastern region (34%) and 22,350 (19%) in the southern region. The distribution of the identified HCV cases was also found to differ between the cities in the same region. In the western region, the largest number of cases was reported in Tripoli (16,560; 31%) followed by Murqub (15,811; 30%) and Jabal al Gharbi (13,219; 25%). There were fewer cases in Sirte (3059; 6%) and Nalut (4660; 9%). In the eastern region, the largest number of cases was in Benghazi (15,932; 41%) followed by Butnan (6640; 17%). In the south, it was highest in Sebha (7493; 34%) followed by Ghat (4426; 20%) and Wadi al Shati (4309; 19%).

**Table 1:** The Distribution of HCV cases over the Libyan regions and districts 2007–2016

Regions and districts	Study period		
	2007–2011	2012–2016	Total n(%)
Eastern region			
Butnan	2520	3020	5540 (14)
Derna	975	1400	2375 (6)
Jabal al Akhdar	1832	3000	4832 (12)
Marj	1320	2100	3420 (9)
Benghazi	6100	6312	12,412 (32)
Al Wahat	2860	3158	6018 (15)
Kufra	1513	3110	4623 (12)
Total	17,120	22,100	39,220 (34)
Western region			
Sirte	699	930	1629 (3)
Misrata	841	1199	2040(4)
Murqub	3401	4059	7460 (14)
Tripoli	7160	8900	16,060 (30)
Jafara	2750	2290	5040 (9)
Zawiya	1419	2150	3569 (7)
Nuqat al Khams	1400	2330	3730 (7)
Jabal al Gharbi	4050	5020	9070 (17)
Nalut	1490	3270	4760 (9)
Total	23,210	30,148	53,358 (46)
Southern region			
Jufra	752	1020	1772 (8)
Wadi al Shati	1190	2119	3309 (15)
Sebha	3222	4011	7233 (32)
Wadi al Hayaa	1320	1860	3180 (14)
Ghat	1316	2150	3466 (16)
Murzuq	1400	1990	3390 (15)
Total	9200	13,150	22,350 (19)
All regions	49,530 (43)	65,398 (57)	114,928

**Fig. 2:**
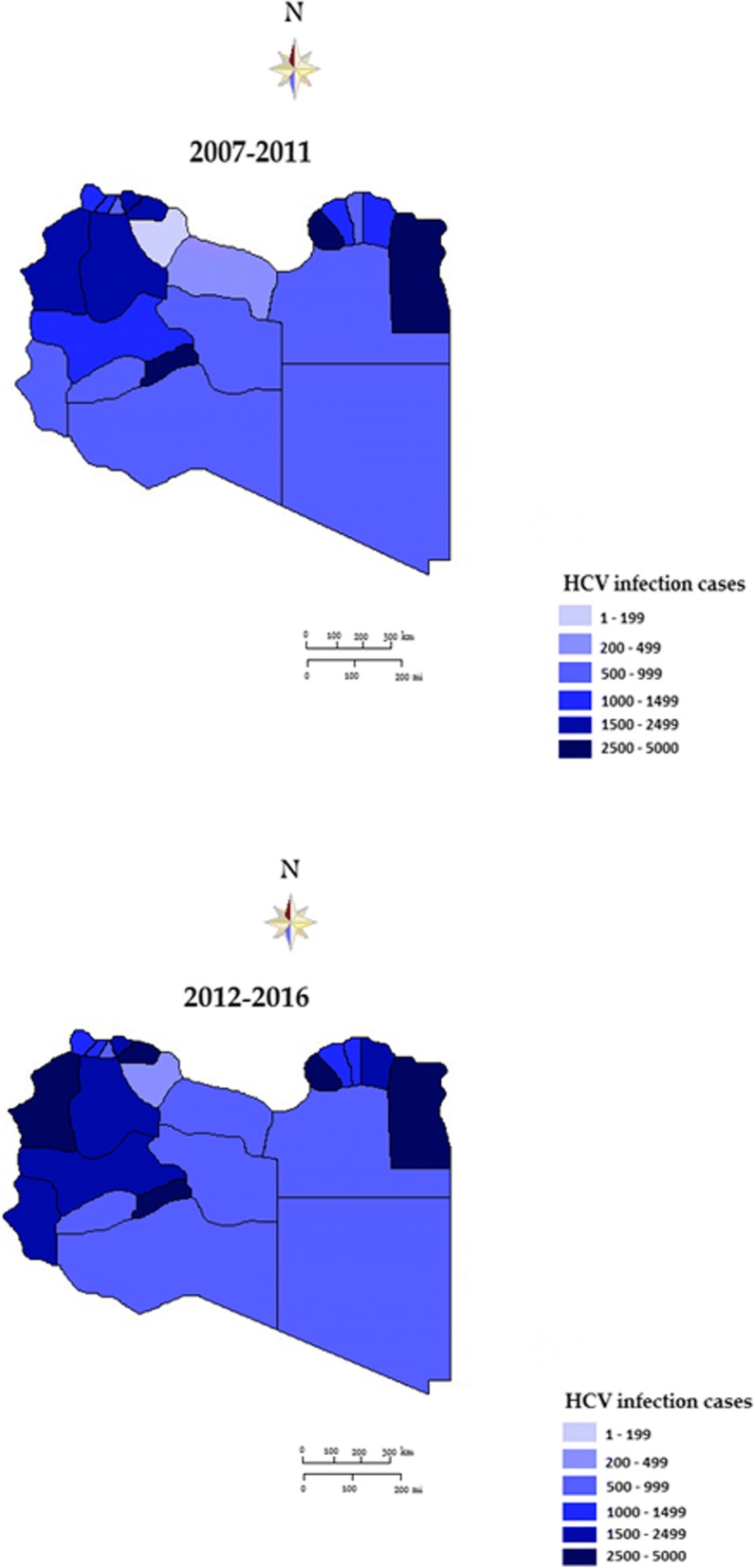
Geo-spatial distributions of identified cases of HCV infection in Libya at district level

Analysis based on continuous images and district-level analysis showed spatiotemporal variation during the ten-year period characterized by an initial expansion and followed by a decline in HCV infection rates. During 2007–2012, the southern region consistently had the highest distribution of HCV, with 5% of the population infected during 2007–2011 (Table 1). The western region had the lowest prevalence rate, particularly in the central districts. HCV density decreased gradually from 2007 till 2011. This was followed by general intensification in the spatial patterns during 2012–2016. In addition, the district distribution of HCV showed persistently high rates in the southern region, particularly in Murzuq (bordering Algeria) and Butnan (bordering Egypt). Figure 2 shows the geographic variability and temporal distribution of HCV infection in each five-year period (Fig. 2a, 2007–2011; b, 2012–2016).

There was clear variability in HCV infection cases at the regional level during the study period. This was more evident in the southern and eastern regions, particularly Sebha, Wadi al Shati and Ghat near the southwestern borders, and Murzuq in the far south. But it was less pronounced in Jufra in the middle of the country, bordering Sert. A similar trend was obvious in districts of the eastern region, with a high rate in Benghazi followed by Butnan on the North-Eastern border. In the Western region, HCV infection rates were stable and without significant variation among the districts. HCV infection rates were very low in the middle part of the country, represented by Jufra, Sirte and Musrata.

**Fig. 3:**
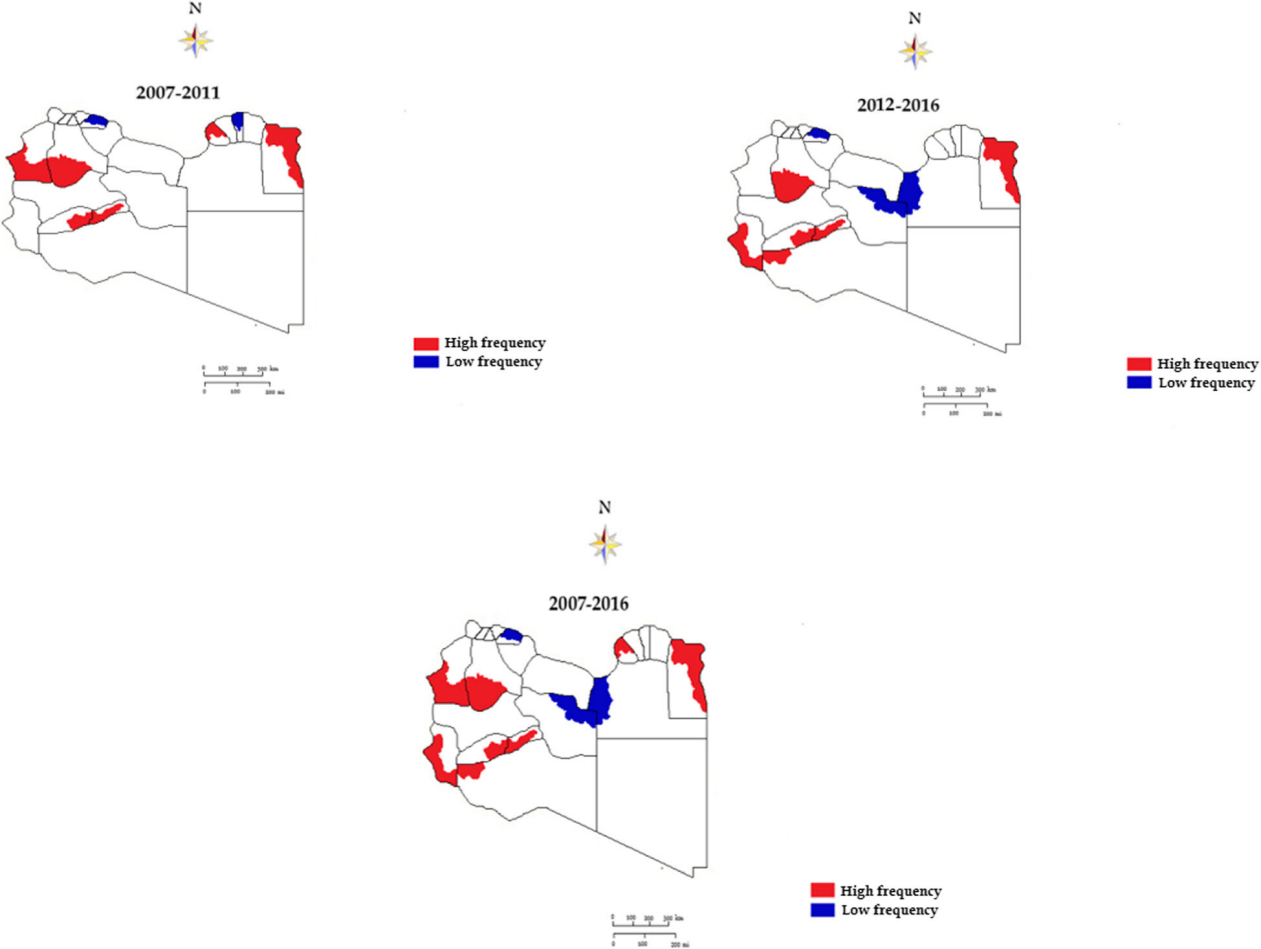
Spatiotemporal patterns of high and low prevalence areas of HCV infection reported during a ten-year study period. **a**, 2007–2011; **b**, 2012–2016; **c**, 2007–2016

Between 2007 and 2016, Moran’s Index generally increased, meaning that the strength of clustering of HCV cases became more prominent. This allowed us to classify the geospatial areas either as a high prevalence area (HPA) or a low prevalence area (LPA) (Fig. 3a, 2007–2011; b, 2012–2016; c, 2007–2016). The high prevalence areas are in districts with a large number of HCV infections with a prevalence rate higher than 1.2% and low prevalence area (LPA- HC) are those of HCV prevalence rate 1–1.2%). This is clearly seen in the eastern region in two districts (Batnan and Benghazi), in four districts in the southern region (Sebha, Wadi al Shati and Ghat) and in Nalout and Al-Jabel Gharbi in western Libya. Table 2 shows the distribution of high prevalence areas (HPA) and low prevalence areas (LPA) of HCV infection between 2007 and 2016. High prevalence areas were observed in Benghazi, Butnan, Sebha, Nalut, Jabal al Gharbi in the first 5 years (2007–2011) and in Butnan, Murzuq, Wadi al Shati, Ghat and Nalut during 2012–2016.

**Table 2:** Distribution of reported high prevalence areas (HPA) and low prevalence area (LPA) of HCV infection in Libya from 2007 to 2016

Year	High frequency area		Low frequency area	
	No.	Location	No.	Location
2007–2008	12	Benghazi, Butnan, Sebha, Jabal al Gharbi	1	Murqub
2009–2010	10	Benghazi, Butnan, Sebha, Nalut, Jabal al Gharbi	1	Murqub, Jabal Alakhdar
2011–2012	9	Benghazi, Butnan, Sebha, Nalut, Jabal al Gharbi	2	Murqub, Al Wahat
2013–2014	14	Butnan, Sebha, Ghat, Jabal al Gharbi	2	Murqub,
2015–2016	17	Butnan, Kufra, Wadi al Shati, Ghat, Nalut	3	Murqub, Al Wahat, Jufra
Total	13	Benghazi, Butnan, Sebha, Nalut, Jabal al Gharbi, Wadi al Shati, Ghat, Kufra	3	Murqub, Al Wahat, Jufra

We also studied the population characteristics and demographics of HCV-infected patients. During 2007–2016, the overall male-to-female ratio was 1:1.33, the mean age of all patients was 47.31 years (95% CI: 47.27–47.35); 39% of them were < 35 years old, and 32% were over 50 years. The relative risk (RR) of HCV infection was compared between males and females. The RR in females compared to males was 1.03 (95% CI: 1.01–1.21) in 2007 and 1.83 (95% CI: 1.42–2.24) in 2016, which is relatively higher during 2012–2016.

Table 3 shows the different demographic factors that could have been involved in HCV infection in the Libyan population during 2007–2016. The socio-demographic determinants included age, sex, education, population density and place of residence. The HCV prevalence rate increased with age. Those aged below 24 years had the lowest prevalence, followed by those 25–35 years old. The reported prevalence was higher among those 36–49 years old but highest in those over 50 years (*P* < 0.001). HCV infection was also higher among males than females aged between 25 and 39 years. The prevalence of HCV was also higher among the illiterate group (37%), in which it ranged from 6 to 25%, depending on the level of education. The place of residence had a significant impact on HCV infection: over 60% of the infections were in urbanized areas (*p* < 0.001). Moreover, the infection rate was higher among unmarried people (39%), those whose living conditions were of a low standard (41%), and those with a minimum income (47%). The impact of the socio-demographic factors on the HCV infection varied from one district to another according to population density, family members and annual income. The high prevalence areas in Benghazi and Tripoli were associated with a high population density, with a population rate over 60 persons/km^2^ with 95%CI 52.95 (51.55–54.34). Although the high prevalence areas in rural districts was contributed with large families size (> 5 members) and low annual income of less than 6500$/year.

**Table 3:** Analysis of demographic characteristics associated with HCV infection in the Libyan population2007–2016

Variables	HCV infection n (%)	Z-test	95% CI	*P* value
Gender				
Male	49,419 (43)	31.23	42.56–43.44	< 0.001 0.001
Female	65,509 (57)	35.833	56.62–57.38	
Age (years)				
< 24	13,791 (12)	35.26	11.46–12.55	0.001
25–35	31,031 (27)	8.136	26.51–27.50	0.001
36–49	33,329 (29)	16.818	26.51–29.49	< 0.001
> 50	36,777 (32)	30.916	31.52–32.48	0.001
Marital status				
Below age	11,493 (10)	26.801	9.46–10.56	0.001 0.001 < 0.001 0.001 0.001
Married	44,822 (39)	100.536	35.55–39.45	
Single	32,180 (28)	35.878	27.51–28.49	
Divorced	10,344 (9)	27.969	8.46–9.57	
Unknown	16,090 (14)	19.027	13.47–14.55	
Education				
Illiterate	42,523 (37)	87.64	35.54–37.46	0.001 0.001 < 0.001 0.001 0.001
Primary	28,732 (25)	21.188	24.5–25.00	
Secondary	20,687 (18)	18.62	21.7–24.91	
Tertiary	6896 (6)	29.065	5.45–6.59	
Unknown	16,090 (14)	19.027	13.47–14.55	
Place of residence				
Urban	70,106 (61)	58.251	60.64–61.36	< 0.001 0.001
Rural	44,822(39)	46.577	38.55–39.45	
Population density				
Low	54,016(47)	13.907	46.58–47.42	0.001 < 0.001
High	60,912(53)	4.398	51.55–54.34	

## Discussion

Medical geospatial analysis has become an important tool for making accurate estimates of the trends in HCV infection epidemics. The number of sero-epidemiological studies of HCV has been increasing, but spatiotemporal studies to detect geographical clusters of the disease are few, particularly in African countries [24]. This study used this technique to analyze the spatiotemporal variation of HCV infection in Libya from 2007 to 2016. The distribution of HCV infections in Libya varied geographically from one region to another and between districts in the same region. The highest prevalence of cases was reported in Sebha, Wadi al Shati, Ghat and Murzuq in the southern region, followed by Benghazi and Butnan in the eastern region, and then Tripoli, Nalut and Jabal al Gharbi in in the western region. A previous national surveillance study has also shown that the sero-prevalence of HCV in Libya varied from 1.2 to 2.2%. The highest prevalence was reported in Sebha (Fezzan) close to sub-Saharan African countries, as well as in Butnan, bordering Egypt [9]. Our results correspond with the findings of a spatial analysis of HCV in China and the Republic of Korea [25, 26], which showed the emergence of an HCV infection epidemic in these countries concentrated in certain provinces and cities. Therefore, HCV prevalence varies not only between countries but also on smaller geographic scales within the same country.

Demographic factors have been found to influence the distribution of HCV infection worldwide. A recent study carried out in Tripoli has shown that the prevalence of HCV, HBV and HIV was associated with intravenous drug use, promiscuity, unemployment, large families and low incomes [27]. Our study is in agreement with this observation: HCV infection rate was higher in the western districts, including Nalut and Jabal al Gharbi, which had large families with poor economic conditions. Further studies are needed to clarify associations with other determinants, including unemployment and drug use.

Butnan and Benghazi in the northeast of Libya had concentrations of HCV cases because they are closer to the Egyptian borders. This is also evident in Murzuq, Wadi al Shati, Sebha and Ghat, which border the sub-Saharan countries of Chad and Niger and have large concentrations of African immigrants. Egypt is the most heavily populated North African nation and has the highest prevalence of HCV in the world. It acts as a main source of labor for the region. Before the 2011 uprising in Libya, there are about two million Egyptians working in Libya, but now there are only about half a million [28]. The prevalence of HCV among these Egyptians reached up to 19% [11]. A recent study carried out in sub-Saharan countries showed that people in that region can move easily around the continent, particularly after the introduction of new regulations by the African Union. Nowadays, there is a massive population exodus from these regions and into Libya, as migrants seek work locally or strive for an expected better life in Europe. Further studies are needed to shed light on the integration and inter-location of HCV infection within neighboring countries of North and sub-Saharan Africa.

These demographic and socio-economic determinants could be used in planning and prevention strategies. Districts where these determinants could point to higher HCV prevalence could be targeted for screening and intervention policies. Similar spatial analyses were carried out in other African countries, such as Malawi, were it was found to be valuable in improving access to healthcare services for HIV and tuberculosis patients [29, 30].

Spatial analysis indicated that hotspots and cold spots existed and were unevenly distributed all over the country. The elevated HCV clusters were located in Benghazi, Butnan, Sebha, Nalut, Jabal al Gharbi, Wadi al Shati, and Ghat. They accounted for over 63% of the identified HCV infection cases during the study period.

In contrast to the changing status of the hotspots and the cold-spots, they were confined to Murqub, Al Wahat and Jufra during the study period. Due to drug-trafficking, the eastern districts bordering Egypt, the western districts bordering Tunisia and Algeria, and the southern districts bordering sub-Saharan countries are considered endemic as a result of HCV. However, drug trafficking poses specific problems for North African countries, and it is exacerbated by the geographical location and the vast area of the region. Morocco is the world’s foremost producer of cannabis resin and remains the main source of the drug for the consumer markets in North Africa and Europe. Studies carried out in North America by Oliveira-Filho et al. have shown that non-injecting drug users have a high HCV infection rate, particularly those who use cannabis and cocaine paste. Hence, this may have an impact on HCV infection in these countries. Further studies are needed to highlight the integration of HCV within North and sub-Saharan countries [31–34]”.

Similar results of local clustering of HCV and other associated infections such as HIV have been reported in South Africa and Italy [35, 36]. Our detection of HCV clusters could be used by healthcare planners in Libya to plan screening and treatment policies. However, it should be noted that not all HCV cases were detected in these clusters. The other cases showed a more random distribution over the whole country. Hence, policies are needed on a national level to cover all the regions and districts [37].

The large sample size and the analysis of geographical distribution and hotspots and cold-spots of the HCV epidemic could be considered important strengths of this study. Uniquely, it goes beyond clustering analysis to map the spatiotemporal variation of HCV risk driven by population-based determinants. It identifies the areas at high risk of HCV infections and thus its consequent complications where public health measures for prevention and treatment could be applied. A modeling system has recently been constructed to predict the prevalence of HCV among risk groups, such as blood donors, in order to propose a preventive strategy in Libya [38]. However, further analysis performed randomly may be needed [5, 39].

### Limitations

Though this study makes an important contribution to advancing the spatiotemporal analysis of HCV infections in Libya, the African continent and developing countries in general, it has some limitations that might have affected the results.

1. Like other studies based on case records and using spatial analysis, some districts may under-report HCV infection cases. Hence, further studies are needed, particularly in the regions of high prevalence of HCV infection.
2. Data collection was based on certain surveillance studies and preserving the confidentiality of the individuals was important. Therefore, it is not always easy to give an accurate timing of HCV infection cases and the precise geographical locations of the patients.
3. Other determinants that were not included in this study may also explain inequalities in HCV infections, such as environmental, social and cultural factors, and access to health care facilities and HCV testing.
4. Data analysis did not take into account the movement of individuals from one region to another within the country, the impact of the variability and the extent of exposure to risk determinants, and the time lag between exposure and detection of HIV infection.

## Conclusion

To the best of our knowledge, this is the first study in North Africa and Arab countries to describe the epidemiological characteristics of HCV infection using geospatial analysis at the country level. We used geospatial analysis to determine geographic and spatiotemporal trends of HCV infection in Libya and highlighted the associated population-based determinants. The results show a distinct variation in the prevalence of HCV infection at regional and district levels in Libya during 2007–2016. This indicates that intervention strategies for HCV prevention should take into account both individual and geographic-area variation. A homogenous national policy may not be effective due to inequalities in HCV prevalence. Understanding the impact of region and district on HCV infection can ensure that intervention is effective in areas with higher infection rates [40, 41]. HCV infections are particularly more prevalent in the southern and eastern districts, but this could have been due to the increasing number of African immigrants in these regions. Hence, further studies are needed at the regional level within North and sub-Saharan countries bordering Libya, particularly Egypt, Sudan, Chad and Niger to shed light on the population-related determinants that may play a role in the upsurge of HCV and other concomitant infections in North Africa [42, 43].

## Abbreviations

HCV: hepatitis C virus
HPA: High prevalence areas
LPA: Low prevalence areas
MI: Moran’s Index
RR: adjusted relative risk
SD: standard deviation
